# Dental Hints Department

**Published:** 1907-08

**Authors:** B. H. Teague

**Affiliations:** Aiken, S. C.


### ...DENTAL HINTS DEPARTMENT...

CONDUCTED BY B. H. TEAGUE, D. D. S., AIKEN, S. C., TO WHOM ALL CONTRIBUTIONS TO THIS DEPARTMENT SHOULD BE SENT

Dr. M. W. White of Yorkome, S. C. in attempting to extract a tooth for a patient pressed so hard against his chair, that he broke one of his ribs.—Newspaper.

Dr. Horace Parker a native of Acworth N. H. after a practice of over sixty years at Edgefield, S. C. died at the latter place at the ripe age of 86 years.

Officers of the South Carolina Dental Association recently elected are T. T. Moore, Jr., Pres., E. J. Etheredge, Vice-Pres., E. M. Kibler, Second Vice-President, W. W. Chisholm, Cor. Secretary, R. Atman Smith, Rec'd Secretary, W. S. Brown, Treasurer.

Hypodermic needles will get dull. Put the wire through the length, and grind the needle, and wire together. The wire can be withdrawn, carrying any bur with it that could clog the needle.—Dr. F. B. Spooner, Summary.

The addition of a fluidrachm of formalin to an ounce of good perfume, sprayed about your office, will keep it from smelling like a "dentist's shop."—Dr. D. D. Campbell, Summary.

Hydronaphthol as a Pulp-Capping.—To avoid the removal of the layer of softened dentin, which, if removed, would probably necessitate the removal of the pulp, mix equal quantities of hydronaphthol and cement, and place the mixture as a capping for the layer of dentin; after allowing it to set proceed with the filling. The hydronaphthol arrests bacterial action.—A. W. McCall, Federal Dental Journal.

Gutta Percha for Filling Teeth.—There is no filling material that will take the place of gutta percha except gold, and there are some places where you cannot put in gold and can put in a good gutta percha filling that will be more serviceable than anything else that could be placed in the cavity.—Review.

Lower Partial Plates With Wire Ccnnection.—In the construction of a lower partial denture I most heartily endorse the use of a wire connecting the two sides. I have been using this for years, and I find it the most satisfactory method of putting in partial plates I ever employed.—F. E. Roach, Review.

Deodorizer.—Tr. Lavender Comp., costs sixty cents per pound and may be diluted to make a gallon or more. A few drops of the diluted solution in your cuspidor will have a pleasant effect on your patients.—H.E.Davis,Dental Review.

Objection to the use of Sulphate of Potassium.—Sulphate of potassium should not be used in plaster that is used for models, for the reason that steam and water attack a model containing sulphate of potassium much more rapidly than if the plaster is mixed without potassium; therefore, we are trying to keep the plaster model hard as long as possible until the rubber has begun to carbonize.—Dr. J. H. Prothero, Dental Register.

Celloidin.—Is acid-free and disolves in alcohol or ether (it is readily soluble in equal parts of both) to a clear, transparent collodion without any sediment. It is non-explosive and the following formula makes an excellent covering for the hands as a substitute for rubber gloves. Celloidin 1 oz., alcohol (96 per cent.) 5 ozs., ether 5 ozs., castor oil (to render the film elastic and flexible) 1-4 to 1-2 oz.—Items of Interest.

The First Permanent Molar.—The dentist is responsible in many cases for decay of the first permanent molars, because so little care is taken of the deciduous teeth that the parents get an idea that they are of little importance and they are not able to differentiate between the first permanent molar and the deciduous molar.—Dr. L. S. Lourie, Review.

Coal oil (kerosene) is a dissolvent of gutta percha and base-plate rubber. It should always be used to smear the parts in repairing a plate with new rubber or vulcanizable gutta percha. It is useful also to moisten a root canal with, when placing a pivot crown with gutta percha.

Action of Tobacco upon the Teeth.—After smoking a Havanna cigar, I made cultures of my own saliva, and found it had the effect of retarding the growth of bacteria more than had any of the mouth-washes I have experimented with. I took the culture just before the cigar, and then repeated every five minutes for one-half hour after smoking.

I am of the opinion that if smokers kept their teeth clean—that is to say, free from foul deposits—and allowed the smoke to percolate around them, they would on an average have much better teeth then have the non-smokers.—F. M. Wells, Dominion Dental Journal.

Shrinkage in Rubber During Vulcanizing.—The amount of shrinkage depends not alone on the time the rubber is subjected to the process of vulcanization, but also upon the temperature. The lower the temperature and steam pressure, the less the loss in shrinkage and the less the contraction in cooling. Low heat and long time also insures an improvement in the texture of the product.—George B. Snow, Dental Brief.

Securing the Fragments of a Lower Fractured Denture.—It is often difficult to securely wax together a broken plate, especially a lower partial. If a strip of wax such as the dental depots use to attach a set of teeth to, is softened and laid on a flat , unyielding surface—a piece of glass—the broken plate may be pressed, teeth down, into the wax, using both hands to hold the pieces in proper position.—A. C. Willman, Review.

Burnishing Gold Plate to a Tooth.—When burnishing a piece of gold plate to a lingual or other aspect of the tooth a good practice is to place a piece of silk ribbon over the gold and carry the ribbon around the tooth so as to have both ends meet on the opposite side and hold them firmly with the fingers, thereby holding the gold plate in place. Under this silk the gold may be burnished with very little trouble.—E. M. S. Fernandez, Review.

Finishing Amalgam Fillings in Interproximal Spaces.—After removing the matrix band draw back and forth a moistened strip of rubber dam as you would sand paper in finishing gold. Do this before the amalgam has set thoroughly and you will have an excellent cervical margin and a surface that will need no further polishing. If rubber is streatched no metal will overhang the margin.—Dr. M. E. Phelps, Summary.

Partial Crowns.—That a partial crown should be used wherever it is possible, instead of a gold banded crown, there can be no doubt. Every conscientious operator seeks to have engrafted upon the roots of molar and bicuspid teeth gold crowns. Wherever possible, he avoids putting a band around the root of the tooth, so that an inlay attachment is a substitute for this, and it is my opinion, after some experience of that kind, that it is firmer than any full gold crown.—E. J. Perry, Review.

Covering Nearly Exposed Pulps.—I am inclined to believe that, given a tooth with a cavity approaching very near to the pulp, the chances of saving that pulp in its vital state are much greater if a layer of some bland material such as gutta-percha or a resin in solution is placed over the region of near exposure, than if the oxphosphate be used exclusively, especially if this be the slow-setting variety with free acid plainly in evidence. Personally, I prefer oxychlorid for covering all nearly exposed pulps, with generally in addition a film of gutta-percha over the region most nearly exposed.—Dr. W. V. Ames.

AMALGAM MIXING.—In many suggestions for mixing amalgam I have read, none are so simple as the use of an ordinary thumb cot, costing five cents at any drug store. I have used this method for four years, and I find it is the easiest and best way to mix a thoroughly incorporated and smooth batch of amalgam.

Expressing the surplus mercury through chamois, the filling need never touch the hands; the cot being sterile leaves it aseptic. It also prevents absorption of mercury by the operator.—DR. C. C. DOBBS, Summary.

REPAIRING GOLD FILLINGS.—My observations have led me to place a great deal of faith in amalgam used in the patching of gold fillings which have failed, either at the cervical margin or the usual proximal margin. There is a peculiar action between gold and amalgam that renders amalgam particularly useful as a patch along-side of gold, and I believe the same holds true of tin, and for the same reason that there is a certain electro chemical action between the base metals of the amalgam or the tin and the gold filling.—W. V. B. AMES, Review.

TO MEND A BROKEN ENGINE CORD.—When your engine cord breaks the common way is to join with thread. This takes time, and is clumsy. Flow sticky wax over the severed ends, and while hot join together, first moistening your fingers. Roll the ends together, allowing them to overlap. Or cut the ends on a slant, bring them together, and see that the wax is very hot, and incorporates with the fibre. This makes a neat, compact joint.—DR. F. B. SPOONER, Summary.

REMOVAL OF PULP WITH CALCIFIC FORMATIONS.—In case of failure to anæsthetize a pulp with cocaine and pressure anæsthesia, due to calcific formation in the pulp, I have obtained good results by the application of sulphuric acid. Make the application for a few minutes, then wipe out the cavity with sodium carbonate and many times you will then be able to anæshetize the pulp.—George W. COOK, Dental Review.

Georgia State Dental Society held its thirty-ninth annual session May 7th and 8th. About two hundred dentists were present. The following were elected as officers for the ensuing year: Dr. W. Crenshaw, Atlanta, president; Dr. T. C. Gibson, Forsyth, first vice-president; Dr. C. P. Davis, Americus, second vice-president; Dr. D. H. McNeill, Athens, corresponding secretary; Dr. D. L. Hill, Atlanta, recording secretary; Dr. H. R. Jewett, Atlanta, treasurer; Dr. H. H. Johnson, Macon, journal editor.

Dental Inspectors Appointed.—The Board of Education of Plainfield, N. J., has granted the request of the New Jersey State Dental Society to introduce into the schools the inspection of children's teeth without expense to the board, and appointed as examiners Dr. William E. Stelle, Dr. O. B. Whitford, and Dr. C. G. Davis.—Plainfield Press.

Dentistry as a profession is young, not yet seventy years of age. Three factors have contributed to its elevation; dental literature, dental colleges, dental associations. Without these three agencies dentistry would be today merely a crude art. We cannot afford to omit any of them. They have been the great fundamental influences which have developed our profession to its present proportions.—Dr. H. A. Smith, Register.

Colson's Poultices.—A little dental apparatus made by Dr. C. B. Colson in Charleston, S. C., I believe, will bring the abscess to a head very quickly, and does draw to a head sometimes and gives relief. It consists of a little emulsive poultice, which, when put into boiling water will swell up and you put that in the mouth between the lip and gum.—Dr. Buttwick, Register.

Gold Sweated Into Matrix.—I use platinum for the matrix. I furthermore use no flux whatever. Nor do I invest. Many suggestions for making porcelain inlays have no application to the making of gold inlays. My conclusion, after much experimenting, is that pure gold "sweated" into the matrix gives the best result. I do not care for a slight tear in the floor of the matrix. By my method of sweating, no gold need pass through to cause a misfit.—Dr. H. Barnes, Register.

"Hand-Me-Down" Crowns.—No one thing today is doing crown and bridge work, that intricate unison of operating room and laboratory, greater harm than these "ready-made, hand-me-down" crown methods; shells made on composite sizes like shoes; seamless system no end; "fit guaranteed and crown on in twenty minutes"; crowns constructed from plaster casts shipped to the wholesale laboratory man who advertises; "Let us do your work while you sleep, six thousand bridges made last year, see our testimonials."—Dr. Clarence J. Grieves, Dental Summary.

Trimming Models.—I am uncompromisingly opposed to the accepted system of scraping or trimming models or impressions, other than putting bead on plate, which will be described in answer to next question. There is always sufficient resistance in soft tissue to float plate if it has been unduly compressed or trimmed away. A better method, which I have never found it necessary to resort to, is to lay tin or lead foil on model where the hard spots are located, but this foil should never extend to the edge of the plate, thus destroying the suction. One can add as many layers of foil as is necessary to suit the case. This foil follows the lines of the mouth and you know definitely how much you have changed it at each point.—Dr. O. H. Simpson, Western.

Blending Colors.—The Sir David Brewster theory was given us tersely. Red, yellow and blue being all the primary colors. Red and yellow produce orange. Blue and yellow produce green. Blue and red produce violet. Tints being made by adding white and shades by the addition of black. Hues by mixing primary colors in varying proportions. Tints by diluting the color or their hues with white. He considered three qualities of color—hue, purity and luminosity. Hue is the chief quality by which one color differs from another.

Purity, the absence of admixture of any other color, or black or white. Luminosity, the strength of light sent to the eye by any color. The most luminous color is yellow, the least is violet.—Amer. D. Jour.

Ranula.—Ranula is a tumor on the floor of the mouth, in form supposed to resemble the belly of a frog. It is sometimes as large or larger than a hickory nut, and has much the physical appearance of the muciparous cyst, except that it is much larger. Formerly ranula was supposed to be caused by stenosis of a salivary duct or by occlusion of the mouth of a duct, but more recent observations indicate that it has no connection with the salivary glands or ducts, but results from closure of the duct from some one of the mucous glands in the floor of the mouth, or the gland of the Bland and Nuhre, under the surface of the tongue.—Era.

Cement For Matrix Moulds.—Use of cement for swadging gold inlay matrices. Secure impression of cavity by using dentalac or modeling compound. Fill this with a stiff cement and let it harden. Insert the cement model thus made in a suitable hand swager and stamp and swage pure gold plate about 36 gauge with soft unvulcanized rubber. Transfer to tooth, and re-burnish if it should not fit accurately. It is then ready to be filled with gold solder to proper contour. If platinum matrix has been used pure, gold solder may be employed. If 22 karat gold matrix is used, a solder that will flow into it without melting the matrix can be used.—Dr. E. C. Sherman, Register.

Eucalyptol in Filling Roots.—In regard to eucalyptol in filling root canals. I can assure you it has given me a great deal of comfort and satisfaction. It is the most saving thing that we can put into root canals, even though we do sometimes force a little medicine through. It leaves the tooth quiet and it stays quiet. In forcing in the gutta-percha point after putting in the eucalyptol I always either apply a hot instrument to that pulp chamber or play hot water over and warm that point and then work it down with a fine point, and I believe I get better results in that way than in trying to force it in the hard condition, depending on the softening of the eucalyptus.—Dr. Wood, Register.

Sea Weed Tents.—This substance can be had of the druggist, and is tough and hard like a piece of rawhide. Cut it into wedges and use to separate the teeth for inlay work. It may be left safely for from 12 to 48 hours. It is especially good where the teeth have approximated each other because of cavities which did not keep the teeth in their normal positions. The tents will swell laterly, or across the grain, two and one-half times their width or thickness. If the cavities are sensitive fill them part full with cement, so as to prevent pressure on the sensitive dentine and pain. The tents may be whittled to any desired form or size. There is no pain from such separation, as the work is done so gradually as to produce no shock.—Dr. E. C. Sherman, Register.

Tumors of the Mouth.—Tumors of the mouth may be benign, but clinical observation indicates that 86 per cent. of all new growths in the mouth are malignant or may become so. It is quite impossible to mention more than a very few of these, and these extremely briefly. Fibroma, sometimes known as spulis, is a fibroid tumor arising from the periosteum. It is found springing up between the teeth, forcing them apart, usually about the anterior teeth. It is pedunculated and sometimes grows to great size. It is thought by some that from irritation it may become malignant. It sometimes breaks down and ulcerates. If it is thoroughly removed early in its growth, together with all of the periosteum connected with it, and a part of the alveolar process, it does not return, but if it is only snipped off or cauterized off it will certainly return.—To temporize with it is dangerous.—Era.

Steadying Sore Teeth.—I have tried taking soft plaster and putting it around the abscessed tooth, so that the plaster is also attached to the other teeth, and I might say making a plaster splint, and after it hardened drill through it to the abscessed tooth cutting through any filling that might be there, and the abscessed tooth suffers no jar that is sufficient to be painful, and one can get into the root canal of an abscessed tooth that way. I have seen it mentioned in the dental journals to use modeling compound in the same way, but it struck me that plaster would hold it firmer. In my experience I have found it to answer very nicely. The greatest trouble with me seems to be to cut into the tooth at all, but after getting it opened up, go ahead and use the broaches and with the escape of gas relief comes.—Dr. E. C. Moore, Register.

Setting an Inlay.—In putting in an inlay instead of putting direct pressure on it, I put it to its place and draw a wide piece of tape over the inlay, the same as though we were polishing a gold filling with polisher. I take a piece of tape a couple of times as wide as the filling, and as you pull it through it will always have some surface of the tape in contact with the filling. Pulling it through that way two or three times wipes off all this surplus that prevents your seeing a real nice margin. At that stage some would put a wedge in. In putting a wedge in they would see a little of that squeeze out, and by putting more pressure on it they might get considerable out, but after you have gotten all out you can possibly get, if you take a piece of tape about one-sixteenth of an inch wide, and rub that over the filling, it does this: It keeps teetering that thing from one end to the other. Keep going over it, and you will be surprised how much closer the inlay will become seated than though you put on any amount of direct pressure.—Dr. W. H. Taggart, Review.

The Approximal Spaces.—The destruction of these spaces is not only a source of great discomfort to the patient, but is positively dangerous. The first danger is that of recurrence of decay. If the teeth are not properly contoured and a proper contact with the approximating tooth has not been made by a small contact point, the margins of the cavity will in all probality fall within the area of susceptibility and as the food and bacteria crowd in between such a beautiful harbor for them, it will not take long to set up a secondary decay that will be far more serious than the first. The second danger is that of gingivitis and pyorrhea, and many teeth have been lost that way, as every man of observation can testify. The gum tissue is crowded out of its place by the teeth falling together, a further irritation is set up by the crowding of food products between the abnormally placed teeth and the end is soon in sight. The third danger, a rare one, but one that does occur, is the growth of gingival tumors as a result of the continual irritation of food debris.—Dr. J. V. Creu, Summary.

Keeping a Tooth Cool While Grinding.—In grinding natural teeth for jacket crowns I invariably keep them dry, directing a jet of compressed air immediately at the point where the stone or bur touches the tooth. By this means I am usually able to grind the most sensitive tooth with little discomfort. But there must be no hesitating or misdirected manipulation. The operator must know in advance precisely the form he wishes to give the tooth, and then go directly to that form. It should never take more than ten, or at most fifteen minutes, to grind down any tooth for a jacket crown. In truing up the walls and making a shoulder at the gingival margin I use sharp fissure burs. And they must be sharp. The moment a stone is not true or a bur is the least dull it is discarded. I have used as many as six burs in preparing a tooth for a crown.—Dr. L. Ladewick, Review.

Base-Plate Material.—Don't throw away your money buying base-plates with which to take the bite, but make a base plate out of your old modeling compound. Have it thick enough to be strong and mold it when hot into your model, with the fingers, and trim. Mold beeswax onto this to get imprint of teeth, etc.

Before putting on your beeswax you can get the sort of relief needed by cutting it in your modeling compound base-plate and noting how it stays up.

After having taken the bite, punch holes through the compound at right angles outlining your air chamber so that when you place it on the model you can run a small, sharp instrument through the holes in the modeling compound base-plate and prick the plaster showing the extremes of the borders of the air chamber, and where it is to be placed on the model.

In difficult mouths you will find this very satisfactory and it will save you many an hour making plates.—L. W. JORDAN, Dental Summary.

AN ANTISEPTIC TOOTH-PASTE.—Chlorate of potash in any strength above 20 per cent will quickly destroy any of the fungi usually found in the mouth, including the germs of putrefaction. A most commendable tooth-paste is this;

| | |
|---|---|
| Chlorate of potash .............. | 50.00 |
| Precipitated chalk.............. | 20.00 |
| Florentine orris root ........... | 15.00 |
| Glycerin ........................ | 15.00 |
| Thymolin ........... ........... | 0.20 |

Mix and make a paste; a little more glycerin may be added if necessary. This not only cleans and whitens the teeth but stimulates the circulation in the gums, arrests fermentation of particles of food left between the teeth, sweetens the breath and leaves a pleasant taste in the mouth.—Clin. Med.

THREE WAYS THAT WILL SURELY CAUSE THE PORCELAIN INLAY TO REMAIN IN PERFECT CONDITION.—Lately, I have used the camphor and also invested my platinum matrices in asbestos, this giving me fifty per cent. betterment in adaption of the inlay. It may take a little more time, but in a few years the patient does not remember whether it took half an hour or three hours; but he does remember whether or not the inlay is *in situ*, and looks well. The finished inlay gave me an absolute fit (it could hardly be dislodged, when tried in) and was one which, when cemented, nothing save tooth fracture could dislodge.

To repeat, Use gum-camphor, invest matrices, also obtain pressure when cementing to place by tying in the inlay, and see the great improvement which will result.—G. B. MITCHELL, Dental Brief

TO REPLACE A BROKEN FACING ON A BRIDGE IN THE MOUTH.—Cut or grind off the old pins even with the backing. Then with a drill the size of the platinum pins drill through the backing at the point of one of the original pins. With a cross-cut fissure bur, drill the backing to the depth of the platinum pins, cut a slot to and including the other pin, and with a cone-shaped bur countersink the slot on the lingual side. Now select a suitable facing, the pins of which will pass into the slot. Remove the facing, and with a flat, wedge-shaped instrument pressed tightly between the pins and close to the porcelain, bend the ends of the pins together, and with a file flatten the pins slightly where the ends meet, so that a small piece of No. 18-k. solder can be laid upon them. Then lay the facing on a piece of asbestos, and with a mouth blowpipe gently heat up and solder the pins together, thus forming a staple, which, when the facing is in place, will enter the slot and extend into the countersunk portion. The latter is then filled with quick-setting amalgam and the repair is completed.—DR. A. J. SAWYER, Dental Cosmos.

